# Eicosapentaenoic Acid Ameliorates Non-Alcoholic Steatohepatitis in a Novel Mouse Model Using Melanocortin 4 Receptor-Deficient Mice

**DOI:** 10.1371/journal.pone.0121528

**Published:** 2015-03-27

**Authors:** Kuniha Konuma, Michiko Itoh, Takayoshi Suganami, Sayaka Kanai, Nobutaka Nakagawa, Takeru Sakai, Hiroyuki Kawano, Mitsuko Hara, Soichi Kojima, Yuichi Izumi, Yoshihiro Ogawa

**Affiliations:** 1 Department of Molecular Endocrinology and Metabolism, Graduate School of Medical and Dental Sciences, Tokyo Medical and Dental University, Tokyo, Japan; 2 Department of Periodontology, Graduate School of Medical and Dental Sciences, Tokyo Medical and Dental University, Tokyo, Japan; 3 Department of Organ Network and Metabolism, Graduate School of Medical and Dental Sciences, Tokyo Medical and Dental University, Tokyo, Japan; 4 Japan Science and Technology Agency, PRESTO, Tokyo, Japan; 5 Development Research, Pharmaceutical Research Center, Mochida Pharmaceutical, Shizuoka, Japan; 6 Micro-Signaling Regulation Technology Unit, RIKEN Center for Life Science Technologies, Wako, Japan; 7 Japan Science and Technology Agency, CREST, Tokyo, Japan; The University of Tokyo, JAPAN

## Abstract

Many attempts have been made to find novel therapeutic strategies for non-alcoholic steatohepatitis (NASH), while their clinical efficacy is unclear. We have recently reported a novel rodent model of NASH using melanocortin 4 receptor-deficient (MC4R-KO) mice, which exhibit the sequence of events that comprise hepatic steatosis, liver fibrosis, and hepatocellular carcinoma with obesity-related phenotypes. In the liver of MC4R-KO mice, there is a unique histological feature termed hepatic crown-like structures (hCLS), where macrophages interact with dead hepatocytes and fibrogenic cells, thereby accelerating inflammation and fibrosis. In this study, we employed MC4R-KO mice to examine the effect of highly purified eicosapentaenoic acid (EPA), a clinically available *n*-3 polyunsaturated fatty acid, on the development of NASH. EPA treatment markedly prevented the development of hepatocyte injury, hCLS formation and liver fibrosis along with lipid accumulation. EPA treatment was also effective even after MC4R-KO mice developed NASH. Intriguingly, improvement of liver fibrosis was accompanied by the reduction of hCLS formation and plasma kallikrein-mediated transforming growth factor-β activation. Moreover, EPA treatment increased the otherwise reduced serum concentrations of adiponectin, an adipocytokine with anti-inflammatory and anti-fibrotic properties. Collectively, EPA treatment effectively prevents the development and progression of NASH in MC4R-KO mice along with amelioration of hepatic steatosis. This study unravels a novel anti-fibrotic mechanism of EPA, thereby suggesting a clinical implication for the treatment of NASH.

## Introduction

Non-alcoholic fatty liver disease (NAFLD) is recognized as a hepatic phenotype of the metabolic syndrome [1]. It encompasses a wide spectrum of liver impairment ranging from benign simple steatosis to non-alcoholic steatohepatitis (NASH), which can lead to cirrhosis and hepatocellular carcinoma [1]. The “two-hit” hypothesis has been proposed as a potential mechanism underlying NASH, in which the first step involves the excessive accumulation of lipids in the liver, thereby sensitizing the liver to the second hits including oxidative stress, lipopolysaccharide, proinflammatory cytokines and adipocytokines [2–4]. However, the precise mechanisms involved in the disease progression from simple steatosis to NASH and hepatocellular carcinoma are still unclear. Accordingly, specific and definitive therapeutic strategies against NASH have not been fully established. It is partly because there are few animal models that reflect the pathophysiology of human NASH.

Recently, we have reported that melanocortin 4 receptor-deficient (MC4R-KO) mice fed high-fat diet develop a liver condition similar to human NASH, which is associated with obesity, insulin resistance and dyslipidemia [5]. MC4R is a seven-transmembrane G protein-coupled receptor that is implicated in the regulation of food intake and body weight [6]. Because MC4R expression is mainly expressed in the hypothalamus and other brain regions [7], it is likely that the hepatic phenotype in MC4R-KO mice results from loss of function of MC4R in the brain, rather than in the liver itself. Accordingly, MC4R-KO mice would provide a novel rodent model with which to investigate the progression from diet-induced hepatic steatosis to NASH. Using this model, we have reported a unique histological structure in the liver termed hepatic crown-like structures (hCLS), in which macrophages surround dead or dying hepatocytes with large lipid droplets [8]. hCLS structurally resembles obesity-induced adipose tissue CLS [8], where sustained interaction between dead adipocytes and macrophages induces adipose tissue inflammation, thereby leading to systemic insulin resistance [9]. Interestingly, the number of hCLS is positively correlated with the extent of liver fibrosis, and myofibroblasts and collagen deposition are observed nearby hCLS [8], suggesting the role of hCLS in the development of NASH. We also detected hCLS in the liver of NAFLD/NASH patients [8]. On the basis of these observations, hCLS may be involved in disease progression from simple steatosis to NASH.

Fish oil rich in *n-3* polyunsaturated fatty acids (PUFAs) such as eicosapentaenoic acid (EPA) or *n-3* PUFAs are clinically effective to treat hypertriglyceridemia. As a molecular mechanism, *n-3* PUFAs improve hepatic lipid metabolism mainly by regulating transcription factors such as peroxisome proliferators-activated receptor α (PPARα) and sterol regulatory element binding protein-1c [10]. In addition, epidemiological and clinical trials have shown that *n-3* PUFAs significantly reduce the incidence of coronary heart disease [11], probably through their pleiotropic effect including an anti-inflammatory property. Given the suppressive effect on hepatic lipid accumulation and inflammation, *n-3* PUFAs could be therapeutically useful to prevent and/or treat NASH. Indeed, recent evidence suggests that *n-3* PUFAs effectively inhibit the development of the diet- or genetically-induced rodent models of NASH, whereas other studies failed [12–17]. However, the recent guideline pointed out that clinical efficacy of *n-3* PUFAs on NAFLD/NASH is controversial [18–25]. Moreover, it is still unclear which species in *n-3* PUFAs are responsible for the treatment of NASH and whether *n-3* PUFAs can regress the hepatic lesion after NASH develops.

In this study, we employed MC4R-KO mice to examine the effect of highly purified EPA on the development of NASH. EPA treatment markedly prevented hepatocyte injury, hCLS formation and collagen deposition along with lipid accumulation in the liver of MC4R-KO mice. Our data also showed that EPA treatment was effective after MC4R-KO mice developed NASH. Intriguingly, the improvement of liver fibrosis was in parallel with the reduction of hCLS formation and hepatocyte injury, suggesting the involvement of hCLS in the beneficial effect of EPA. Collectively, this study raises a novel anti-fibrotic mechanism of EPA in a mouse model of NASH, thereby suggesting its therapeutic efficacy in NASH.

## Methods

### Materials

Preparation and characterization of highly purified EPA ethyl ester (purity: >98%, Mochida Pharmaceutical Co., Ltd., Tokyo, Japan) used in animal studies were reported elsewhere [26,27]. Ethyl palmitate (purity > 95%) was purchased from Wako (Tokyo, Japan).

### Animals

The MC4R-KO mice on the C57BL/6J background were a generous gift from Dr. Joel K. Elmquist (University of Texas Southwestern Medical Center) [6]. Male C57BL/6J wildtype mice were purchased from CLEA Japan (Tokyo, Japan). The animals were housed in individual cages in a temperature-, humidity- and light-controlled room (12-h light and 12-h dark cycle) and allowed free access to water and standard diet (SD) (CE-2; CLEA Japan). After 1-week acclimation period, 8 week-old male mice were given free access to water and either SD or Western diet (WD) (D12079B; Research Diets, New Brunswick, NJ) supplemented with 5% (wt/wt) ethyl palmitate or EPA ethyl ester [27]. Detailed dietary composition of the SD and WD is shown in S1 Table. All diets were changed every day and served with a non-metallic feeder to prevent oxidization of fatty acids. In this study, we conducted two experimental protocols to evaluate the preventive and therapeutic effect of EPA, *i*.*e*. EPA treatment throughout the experimental period (24 weeks) and 4-week EPA treatment after the development of NASH, respectively. At the end of the experiments, they were sacrificed, when fed *ad libitum*, under intraperitoneal pentobarbital anesthesia (30 mg/kg). All animal experiments were conducted in accordance to the guidelines for the care and use of laboratory animals of Tokyo Medical and Dental University. The protocol was approved by Tokyo Medical and Dental University Committee on Animal Research (No. 0140016A, No. 2011-207C3).

### Blood Analysis

Blood glucose levels were measured by the blood glucose test meter (Glutest PRO R; Sanwa-Kagaku, Nagoya Japan). Serum concentrations of alanine aminotransferase (ALT), triglyceride (TG), free fatty acid (FFA) and total cholesterol (TC) were measured by the respective standard enzymatic assays. Serum concentrations of adipocytokines were determined by the commercially available enzyme-linked immunosorbent assay (ELISA) kits (insulin: Morinaga, Tokyo, Japan; adiponectin: Otsuka Pharmaceutical, Tokyo, Japan; leptin: R&D systems, Minneapolis, MN). For insulin tolerance test, 1-hour fasted mice injected intraperitoneally with human insulin at 1.0 U/kg and blood glucose levels were determined before and at 15, 30, 60, 90 and 120 min after insulin administration.

### Hepatic TG Content

Total lipids in the liver were extracted with ice-cold 2:1 (vol/vol) chloroform/methanol. The TG concentrations were measured by an enzymatic assay kit (Wako Pure Chemicals, Osaka, Japan) [5].

### Quantification of Active TGFβ1 Content

Active transforming growth factor-β1 (TGFβ1) protein levels in the liver were measured as described [28]. Briefly, frozen liver samples were homogenizes in a lysis buffer (20 mM Tris, pH 7.5, 10 mM ethylenediaminetera-acetic acid) supplemented with protease inhibitors (2 mM phenylmethane sulfonyl fluoride, 0.5 mM dithiothreitol, protease inhibitor cocktail (Sigma, St. Louis, MO)). Samples were centrifuged at 17,000 x g for 20 min at 4°C and the supernatants were subjected to the ELISA kit for mouse TGFβ1 (R&D). Active TGFβ1 protein levels were normalized to the protein concentrations.

### Histological Analysis

The liver samples were fixed with neutral-buffered formalin and embedded in paraffin. Four-μm-thick sections were stained with Masson-trichrome and Sirius red [5]. The presence of F4/80-positive macrophages was detected immunohistochemically using the rat monoclonal anti-mouse F4/80 antibody described elsewhere [29]. Proteolytic activation of latent TGFβ was detected with antibody against R58 latency associated protein degradation products (LAP-DPs) [30]. Apoptotic cells were detected by TdT mediated dUTP-biotin nick end labeling (TUNEL) assay using ApopTag Plus Peroxidase In Situ Apoptosis Detection Kit (Millipore, Billerica, MA). The Sirius red-positive and R58 LAP-DP-positive areas were measured using the software WinROOF (Mitani, Chiba, Japan). TUNEL-positive cells were counted in the whole area of each section and expressed as the mean number/mm^2^. The liver histology was assessed by two investigators without knowledge of the origin of the slides according to the NASH clinical research network scoring system [31].

### Quantitative Real-Time PCR

Total RNA was extracted from the liver using Sepasol reagent (Nacalai Tesque, Kyoto, Japan). Quantitative real-time PCR was performed with StepOnePlus Real-time PCR System using Fast SYBR Green Master Mix Reagent (Applied Biosystems, Foster City, CA) as described previously [5]. Primers used in this study were described in S2 Table. Levels of mRNA were normalized to those of 36B4 mRNA.

### Statistical Analysis

Data are presented as mean ± SE, and *P* < 0.05 was considered statistically significant. Statistical analysis was performed using analysis of variance followed by Scheffe’s test. Differences between two groups were compared using Student *t*-test. Pearson correlation coefficient was employed to investigate the correlation among the numbers of hCLS and TUNEL-positive cells, and the extent of fibrosis.

## Results

### Preventive effect of EPA on hepatic lipid accumulation in MC4R-KO mice

**Fig 1:**
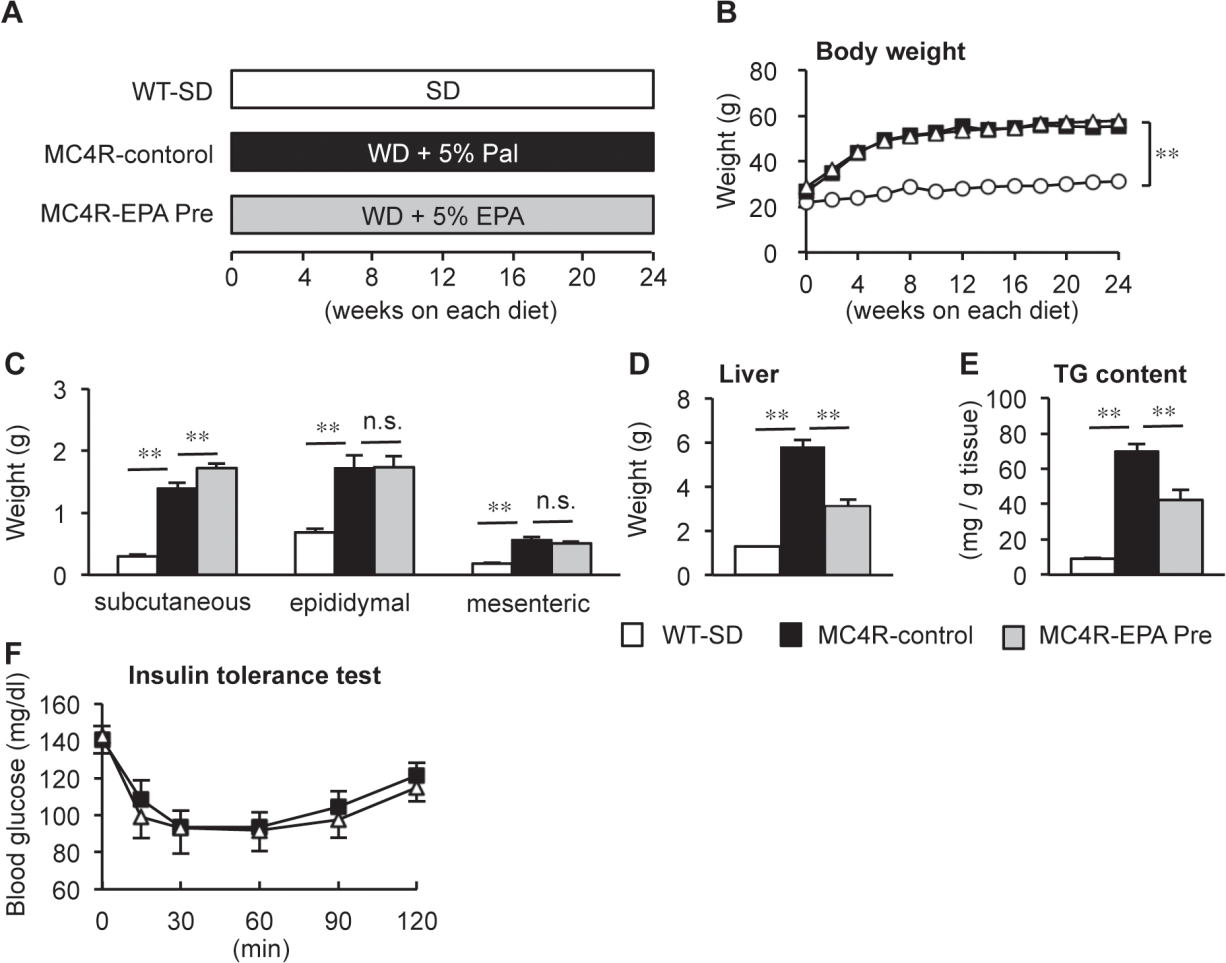
Body weight and tissue weights in MC4R-KO mice treated with EPA for 24 weeks. (A) Experimental protocol of preventive EPA treatment. Growth curve (B) and weights of the subcutaneous, epididymal and mesenteric white adipose tissues (C) and liver (D) of male MC4R-KO (MC4R) and wildtype (WT) mice. WT-SD, WT mice fed standard diet (SD); MC4R-control, MC4R-KO mice fed Western diet (WD) supplemented with 5% (wt/wt) palmitate; MC4R-EPA Pre, MC4R-KO mice fed WD supplemented with 5% (wt/wt) EPA for 24 weeks. Open circle, WT-SD; Open triangle, MC4R-control; closed square, MC4R-EPA Pre. (E) Liver triglyceride (TG) content at 24 weeks. (F) Insulin tolerance test (ITT) at 12-week WD feeding. Open triangle, MC4R-control; closed square, MC4R-EPA Pre. ** *P* < 0.01; n.s., not significant. WT-SD, *n* = 8; MC4R-control, *n* = 7; MC4R-EPA Pre, *n* = 10.

First, we examined whether EPA treatment prevents the development of NASH using our mouse model of NASH. Wildtype mice were fed SD (WT-SD) and MC4R-KO mice were fed control diet, WD plus 5% weight palmitate (MC4R-control) or with the diet, in which 5% weight palmitate was replaced to EPA (MC4R-EPA Pre) for 24 weeks (Fig 1A). The amount of food intake was comparable between control and EPA-treated MC4R-KO mice (data not shown). The MC4R-KO mice fed control diet showed accelerated body weight gain relative to wildtype mice fed SD, along with increased weights of adipose tissue and liver (Fig 1B–1D) as reported [5,8]. EPA treatment showed no appreciable or only marginal effect on body weight and adipose tissue weights (Fig 1B and 1C). On the other hand, the liver weight and the hepatic TG content were markedly reduced in EPA-treated MC4R-KO mice relative to control MC4R-KO mice (*P* < 0.01, Fig 1D and 1E). Hepatic fatty acid composition analysis revealed increased hepatic EPA content and decreased arachidonic acid content (S3 Table). EPA treatment also reduced serum concentrations of TC, FFA, and ALT in MC4R-KO mice, whereas EPA treatment did not affect glucose metabolism and insulin resistance (Table 1, Fig 1F). Since unbalanced production of pro- and anti-inflammatory adipocytokines in obesity has been implicated in the pathogenesis of NASH [32], we examined serum adipocytokine concentrations and found that EPA treatment significantly increased serum adiponectin concentrations in MC4R-KO mice (Table 1). On the other hand, EPA treatment did not affect serum concentrations of leptin in MC4R-KO mice (Table 1).

**Table 1:** Serological parameters of MC4R-KO and WT mice treated with EPA for 24 weeks.

	WT	MC4R-KO	
	SD	Control	EPA-pre
BG (*ad lib*, mg/dL)	144.0 ± 7.3	134.3 ± 9.7	119.1 ± 6.2
Insulin (*ad lib*, ng/mL)	0.6 ± 0.1	4.3 ± 1.3**	3.7 ± 1.8
TG (mg/dL)	57.1 ± 30.9	30.9 ± 3.2**	34.6 ± 6.0
TC (mg/dL)	70.8 ± 3.0	290.1 ± 15.0**	122.5 ± 8.3^¶^
FFA (mEq/L)	1.13 ± 0.10	1.33 ± 0.09	0.80 ± 0.05^¶^
ALT (IU/L)	47.5 ± 5.6	539.1 ± 87.2**	120.5 ± 16.8^¶^
Adiponectin (μg/mL)	12.7 ± 1.4	7.8 ± 1.0	17.7 ± 2.0^¶^
Leptin (ng/mL)	8.7 ± 1.8	114.3 ± 4.5**	119.9 ± 8.9

WT, wildtype; SD, standard diet; BG, blood glucose; TG, triglyceride; FFA, free fatty acid; TC, total cholesterol; ALT, alanine aminotransferase. Data are expressed as the mean ± SE.

**P < 0.01 vs. WT-SD

^¶^ *P* < 0.01 vs. MC4R-Control. *n* = 7–10

### Effect of EPA on the development of liver fibrosis in MC4R-KO mice

**Fig 2:**
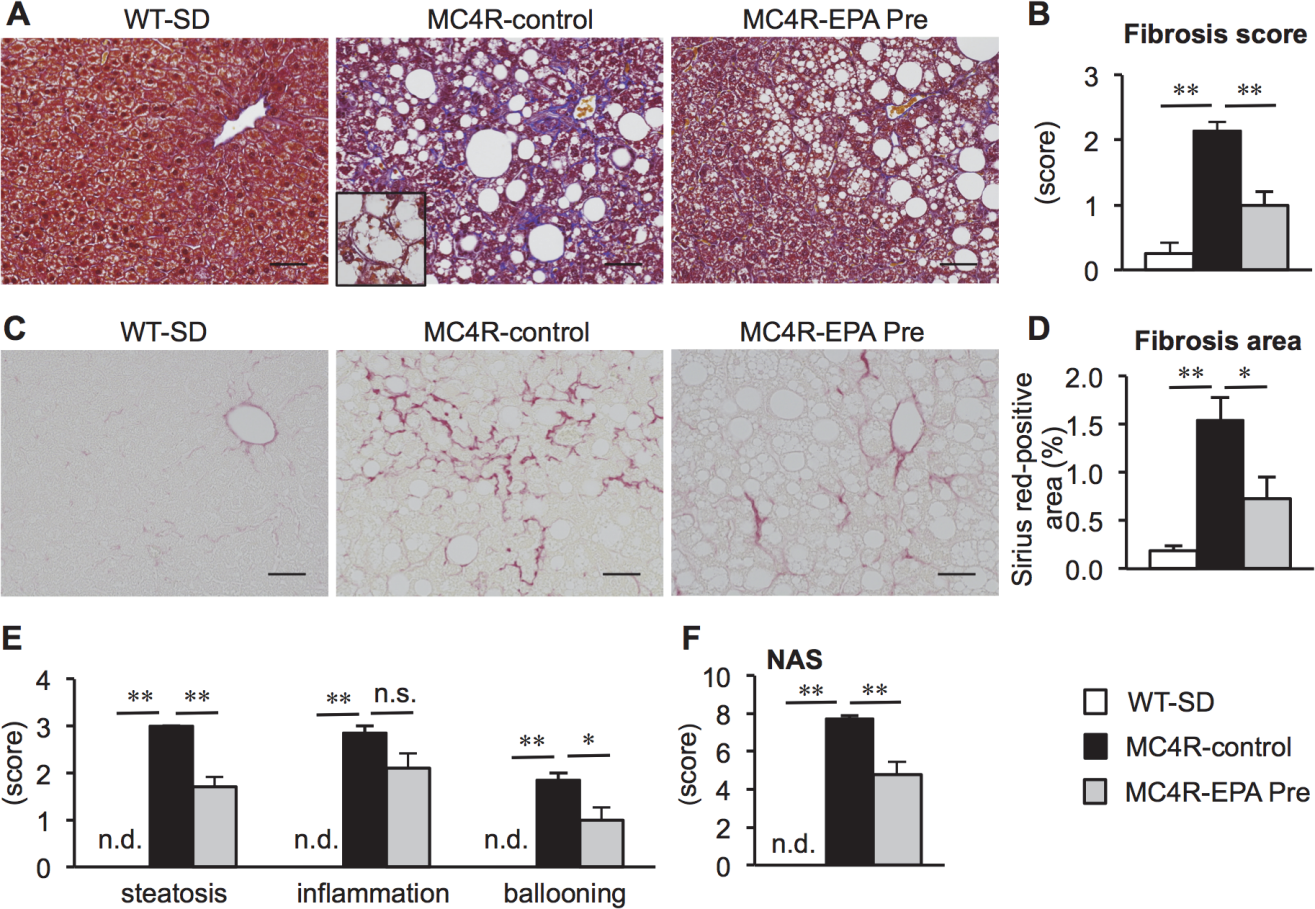
Effect of EPA on liver injury and fibrosis in MC4R-KO mice. Fibrillar collagen deposition evaluated by Masson-trichrome staining (A) and fibrosis scores (B) at 24 weeks. Inset: Representative image of hepatocyte ballooning. Sirius red staining (C) and quantification of Sirius red-positive area (D). Scores of steatosis, lobular inflammation, ballooning degeneration (E) and non-alcoholic fatty liver disease activity score (NAS) (F). Scale bars, 50 μm. * *P* < 0.05; ** *P* < 0.01; n.s., not significant; n.d., not detected. WT-SD, *n* = 8; MC4R-control, *n* = 7; MC4R-EPA Pre, *n* = 10.

**Fig 3:**
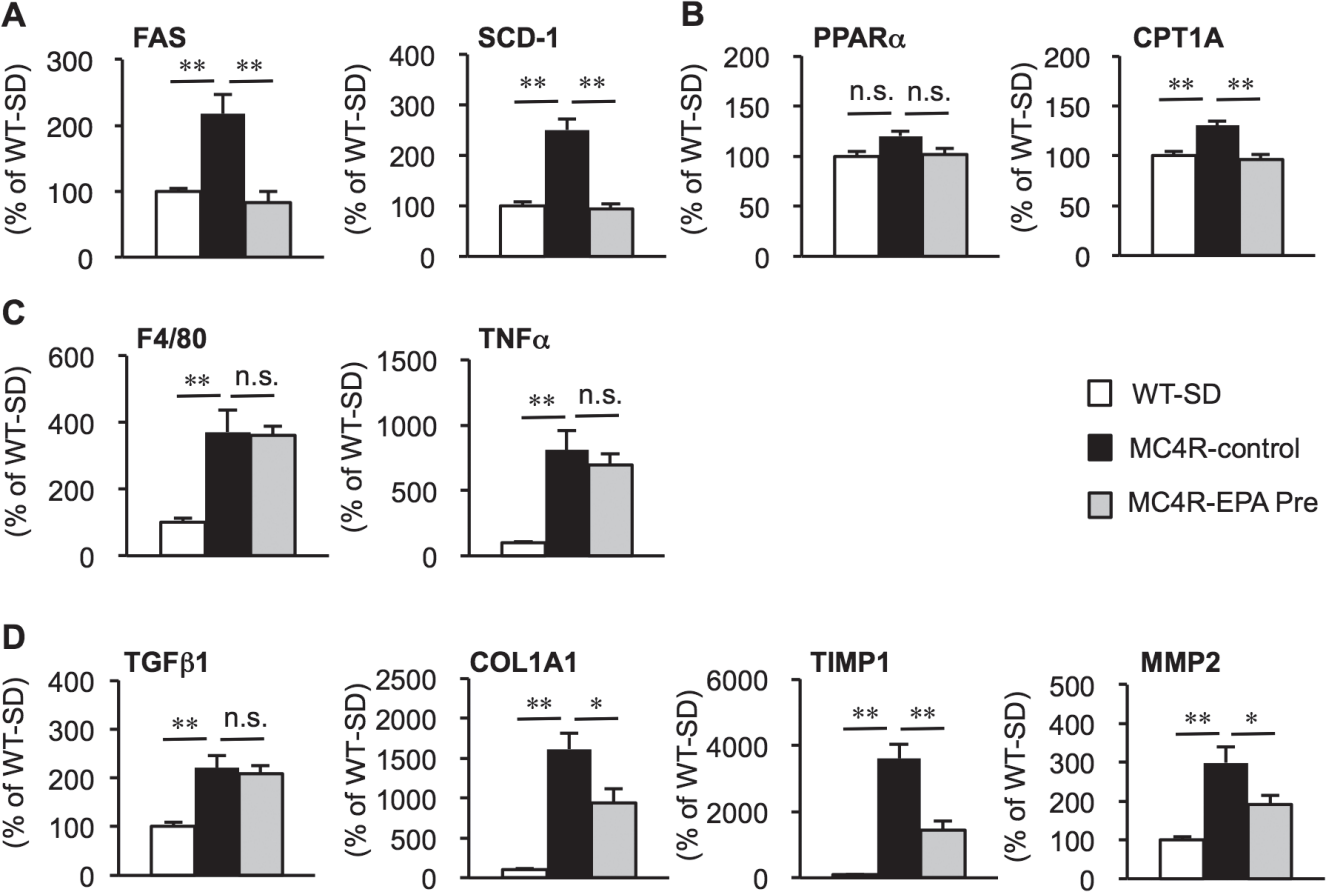
Effect of EPA on hepatic mRNA expression in MC4R-KO mice. Hepatic mRNA expression levels after 24 weeks of EPA treatment. mRNA expression of genes related to *de novo* lipogenesis (fatty acid synthase (FAS) and stearoyl-CoA desaturase (SCD-1)) (A), β-oxidation (peroxisome proliferators-activated receptor α PPARα and carnitine palmitoyltransferase 1A (CTP-1A)) (B), inflammatory markers (F4/80 and tumor necrosis factor α (TNFα) (C) and fibrogenic factors (transforming growth factor β1 (TGFβ1), collagen α1(I) (COL1A1), tissue inhibitor of metalloproteinase-1 (TIMP1) and matrix metalloproteinase-2 (MMP2)) (D). * *P* < 0.05; ** *P* < 0.01; n.s., not significant. WT-SD, *n* = 8; MC4R-control, *n* = 7; MC4R-EPA Pre, *n* = 10.

After 24 weeks, the livers from MC4R-KO mice fed control diet exhibited micro- and macrovesicular steatosis, ballooning degeneration, massive infiltration of inflammatory cells and pericellular fibrosis (Fig 2A and 2C) as reported previously [5,8]. On the other hand, steatotic changes and ballooning degeneration were markedly suppressed in EPA-treated MC4R-KO mice (Fig 2A and 2C). The fibrosis score and fibrosis area were also significantly decreased by EPA treatment (Fig 2B and 2D). Although the inflammation score was unchanged, the scores for steatosis and ballooning degeneration were decreased by EPA treatment, so that there was a significant reduction in NAS in EPA-treated MC4R-KO mice relative to control MC4R-KO mice (Fig 2E and 2F). In this study, mRNA expression of genes related to *de novo* lipogenesis (fatty acid synthase (FAS) and stearoyl-CoA desaturase-1 (SCD-1)) and β-oxidation (carnitine palmitoyltransferase 1A (CPT1A)) was markedly increased in the liver of control MC4R-KO mice relative to wildtype mice as reported [5], which was significantly suppressed by EPA treatment (Fig 3A and 3B). There was no apparent change in mRNA expression of proinflammatory genes such as (macrophage marker F4/80 and tumor necrosis factor-α (TNFα)) (Fig 3C). On the other hand, mRNA expression of TGFβ1-target genes such as collagen α1(I) (COL1A1), tissue inhibitor of metalloproteinase-1 (TIMP1) and matrix metalloproteinase-2 (MMP2) was significantly suppressed, although EPA treatment did not affect mRNA expression of TGFβ1 (Fig 3D). These observations, taken together, suggest that EPA treatment effectively prevents the development of liver fibrosis in MC4R-KO mice.

### Effect of EPA on hCLS formation and hepatocyte apoptosis in MC4R-KO mice

**Fig 4:**
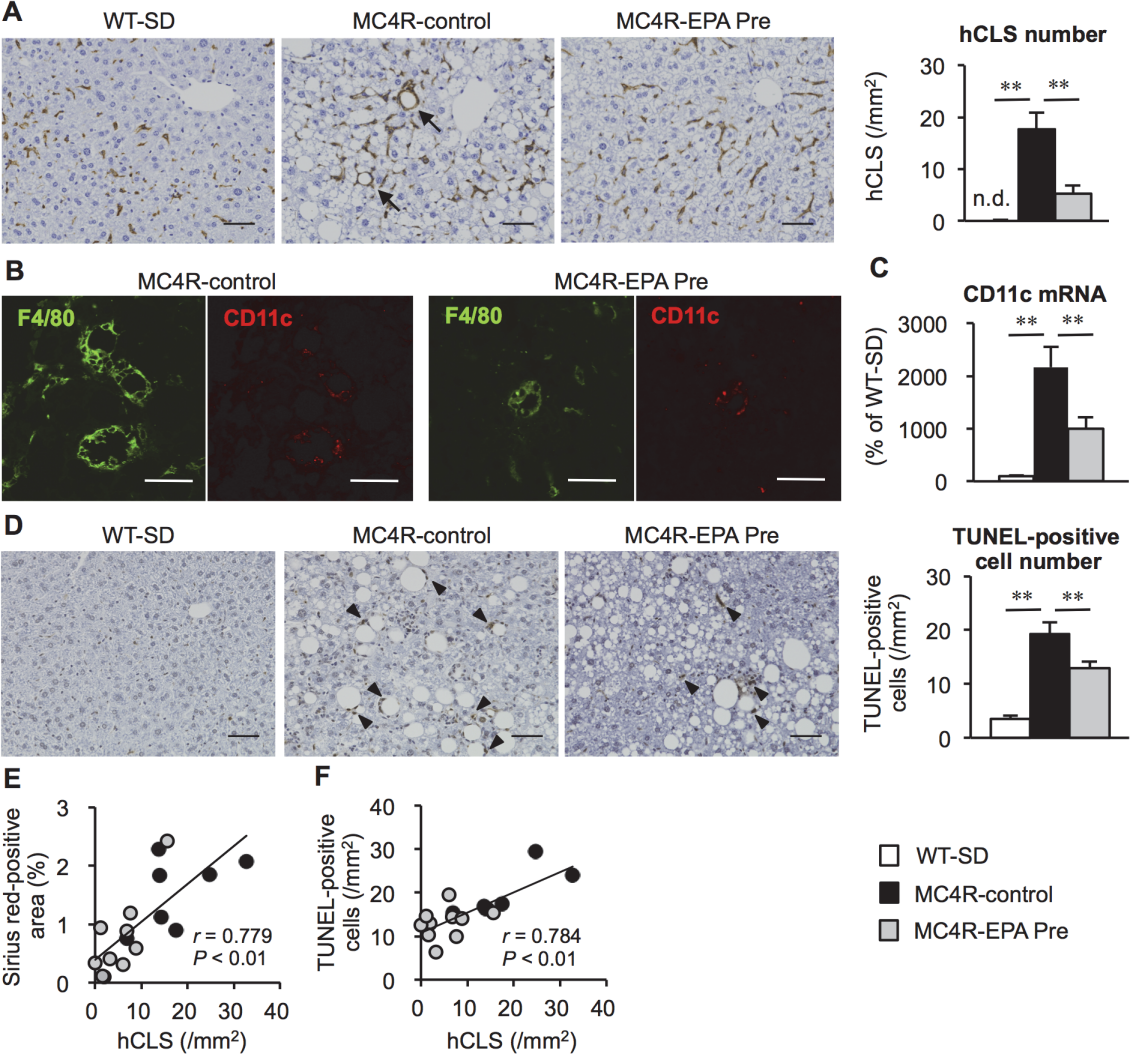
Effect of EPA on hCLS formation and apoptosis in the liver of MC4R-KO mice. (A) F4/80 immunostaining of the liver after 24 weeks of EPA treatment. Arrows indicate characteristic histological features termed “hepatic crown-like structures (hCLS)”. (B) Immunofluorescent staining for F4/80 (green) and CD11c (red). (C) Hepatic mRNA expression of CD11c. (D) TdT mediated dUTP-biotin nick end labeling (TUNEL) immunostaining and the number of TUNEL-positive cells. Arrowheads indicate TUNEL-positive cells. Correlation of the number of hCLS and the fibrosis area (E) and the number of TUNEL-positive cells (F). Scale bars, 50 μm. * *P* < 0.05; ** *P* < 0.01; n.d., not detected. WT-SD, *n* = 8; MC4R-control, *n* = 7; MC4R-EPA Pre, *n* = 10.

We have recently reported a unique histological structure or hCLS in the liver of MC4R-KO mice, where dead hepatocytes are surrounded by CD11c-positive macrophages [8]. Our data also suggest that hCLS promotes liver fibrosis during the progression from simple steatosis to NASH [8]. We found that EPA treatment effectively suppresses hCLS formation in MC4R-KO mice (Fig 4A), In this study, the F4/80-positive area was roughly comparable between the treatments (data not shown). Double immunofluorescent staining of F4/80 and CD11c revealed that hCLS-constituting macrophages are positive for CD11c in EPA-treated MC4R-KO mice (Fig 4B), whereas hepatic mRNA expression of CD11c was significantly suppressed in EPA-treated MC4R-KO mice (Fig 4C) in parallel with reduced number of hCLS. Since hCLS-constituting macrophages are considered to engulf dead hepatocytes and residual lipids [33], we examined apoptotic cells by TUNEL staining. Compared to SD-fed wildtype mice, control MC4R-KO mice showed marked increase in the number of TUNEL-positive cells, most of which assembled around large lipid droplets (Fig 4D). TUNEL-positive cells were decreased in number in EPA-treated MC4R-KO mice relative to control MC4R-KO mice (Fig 4D), which was in parallel with serum ALT concentrations (Table 1). In this study, the number of hCLS was positively correlated with that of TUNEL-positive cells as well as the extent of liver fibrosis (Fig 4E and 4F) [8]. Collectively, these observations suggest that EPA suppresses hepatocyte apoptosis in MC4R-KO mice, which may prevent hCLS formation and fibrotic changes.

### Effect of EPA on hepatic TGFβ activation in MC4R-KO mice

**Fig 5:**
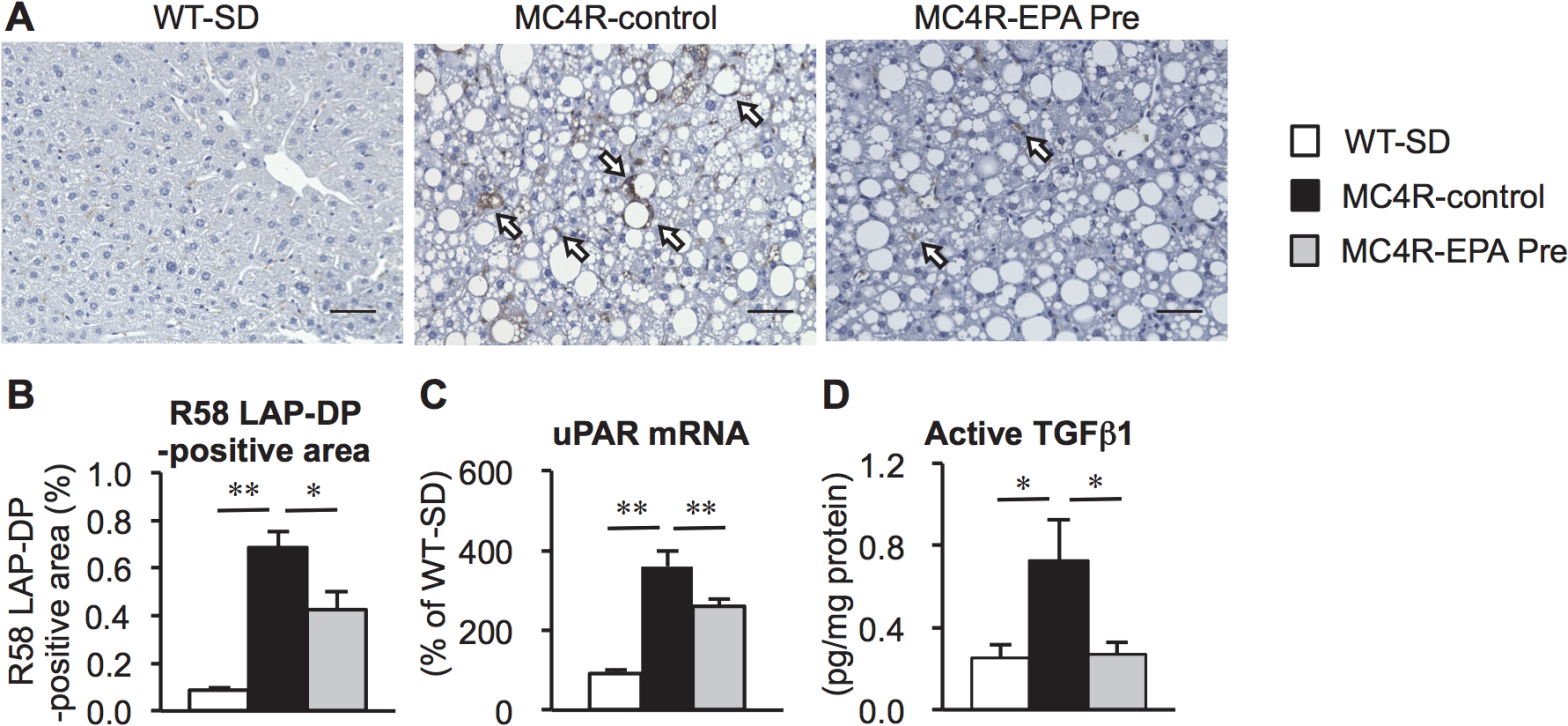
Effect of EPA on hepatic TGFβ activation in MC4R-KO mice. (A) Immmunostaining with anti-R58 LAP-DP antibody to determine TGFβ activation in the liver after 24 weeks of EPA treatment. (B) Quantification of the R58 LAP-DP-positive area. (C) Hepatic mRNA expression of urokinase-type plasminogen activator receptor (uPAR). (D) Active TGFβ protein levels in the liver. Scale bars, 50 μm. * *P* < 0.05; ** *P* < 0.01. WT-SD, *n* = 8; MC4R-control, *n* = 7; MC4R-EPA Pre, *n* = 10.

Since there was no difference in hepatic TGFβ mRNA expression in MC4R-KO mice between the treatments, we next investigated the TGFβ activation state in the liver. We performed immunostaining using the anti-R58 LAP-DP antibody, which can detect the cleavage site of LAP, serving as a foot print for generation of active TGFβ [30]. The R58 LAP-DP-positive area was increased in the liver of control MC4R-KO mice relative to wildtype mice, which was decreased by EPA treatment (Fig 5A and 5B). The latent TGFβ is activated by plasma kallikrein that is bound to urokinase-type plasminogen activator receptor (uPAR) on the cell surface [34]. In this study, mRNA expression of uPAR was significantly decreased in EPA-treated MC4R-KO mice (Fig 5C). We also confirmed the decreased protein levels of active TGFβ in the liver of EPA-treated MC4R-KO mice (Fig 5D). These observations suggest that EPA suppresses TGFβ activation, thereby inhibiting disease progression from simple steatosis to NASH.

### Therapeutic effect of EPA on the progression of NASH in MC4R-KO mice

**Fig 6:**
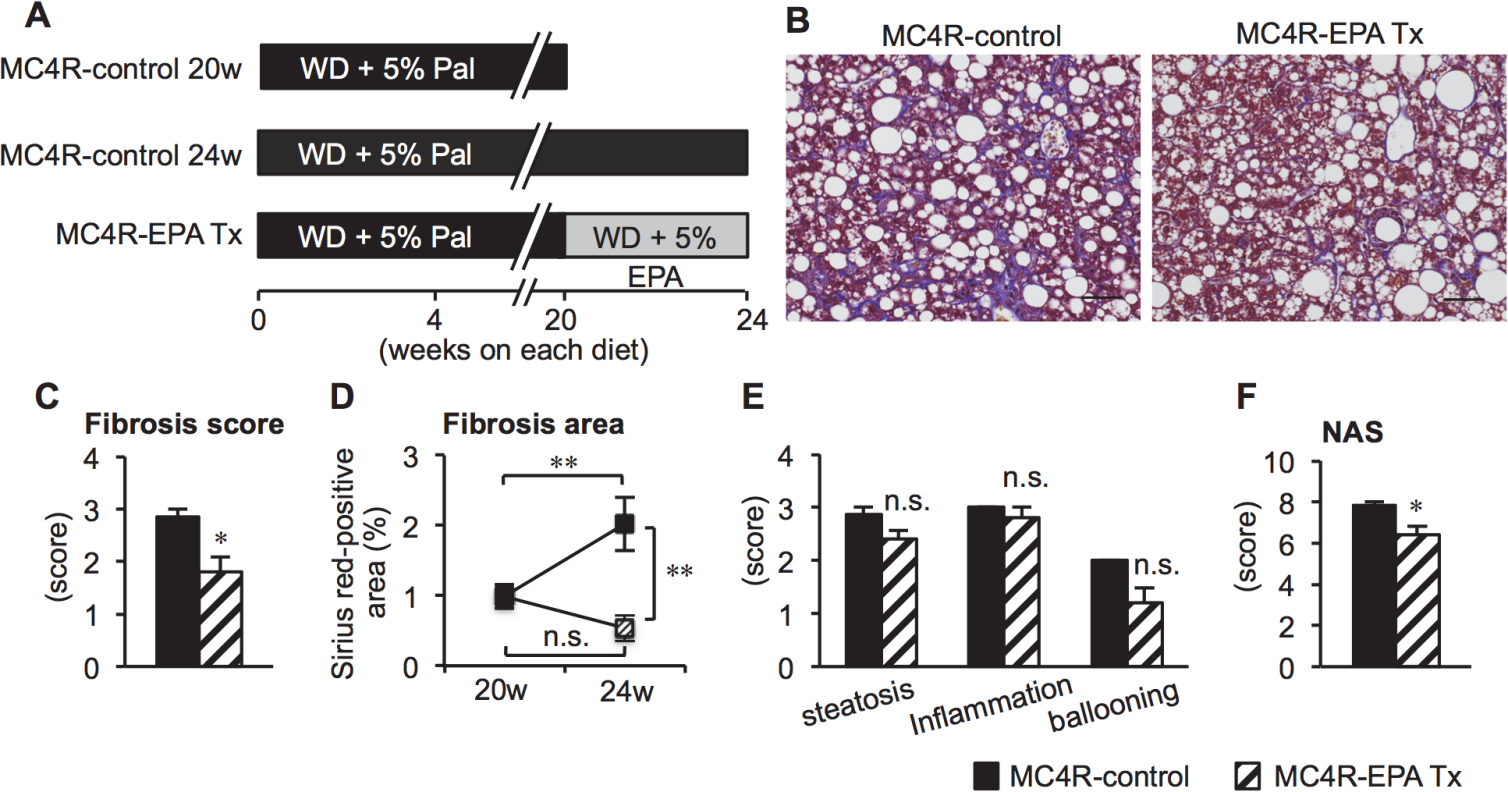
Histological analysis of the liver of MC4R-KO mice treated with EPA for 4 weeks after the development of NASH. (A) Experimental protocol of therapeutic EPA treatment. Fibrillar collagen deposition evaluated by Masson-trichrome staining (B) and fibrosis scores (C). (D) Quantification of Sirius red-positive area. Scores of steatosis, lobular inflammation, ballooning degeneration (E) and NAS (F). Scale bars, 50 μm. * *P* < 0.05; ** *P* < 0.01; n.s., not significant. MC4R-control, *n* = 7; MC4R-EPA Pre, *n* = 10.

We also examined whether EPA treatment is effective after 20 weeks of control diet feeding when MC4R-KO mice develop NASH [8]. In this study, MC4R-KO mice were fed either control diet (MC4R-KO control) or EPA-supplemented diet (MC4R-EPA Tx) for another 4 weeks (Fig 6A). The liver weight and hepatic TG content were significantly reduced in EPA-treated MC4R-KO mice relative to control MC4R-KO mice at 24 weeks, whereas body weight and adipose tissue weight except for the epididymal fat depot were unchanged between the groups (S1A–S1D Fig.). Serum concentrations of TC and ALT were reduced, and those of adiponectin were increased in EPA-treated MC4R-KO mice (S4 Table). In this study, histological analysis revealed that EPA treatment for 4 weeks significantly suppressed the progression of liver fibrosis in MC4R-KO mice (Fig 6B–6D). Moreover, the NAS was significantly decreased in EPA-treated MC4R-KO mice relative to control MC4R-KO mice, although the change in each NAS component (steatosis, inflammation, and ballooning degeneration) did not reach statistic significance (Fig 6E and 6F). Similar to the preventive protocol, hepatic mRNA expression of genes related to *de novo* lipogenesis, β-oxidation, and fibrogenesis was decreased in EPA-treated MC4R-KO mice relative to control MC4R-KO mice (S2 Fig.). The number of hCLS was also significantly reduced in EPA-treated MC4R-KO mice relative to control MC4R-KO mice, along with down-regulation of CD11c mRNA expression (S3A–S3D Fig.). Furthermore, EPA treatment resulted in a significant reduction in the number of TUNEL-positive cells and TGFβ activation (S3E–S3H Fig.). These observations, taken together, suggest that EPA suppressed the progression of liver fibrosis in MC4R-KO mice after the mice developed NASH.

## Discussion

Using a variety of animal models through genetic, dietary, and/or pharmacologic approaches, many attempts have been made to identify novel therapeutic strategies for NASH, while their clinical efficacy is still unclear. It is partly because of the limited availability of appropriate animal models that reflect a liver condition of human NASH [35]. For instance, dietary deficiency of methionine and choline develops steatosis and mild fibrosis in the liver, although without obesity and insulin resistance [35]. Since NASH is considered as the hepatic phenotype of the metabolic syndrome, crosstalk among multiple organs should be involved in the pathophysiology of NASH. In this regard, MC4R-KO mice, a unique rodent model of NASH accompanied by obesity and systemic insulin resistance, would be useful for evaluating the effectiveness of novel drugs to treat NASH. This is the first report to evaluate drug efficacy using MC4R-KO mice. In this study, we demonstrate that EPA treatment effectively suppresses the development and progression of liver fibrosis along with marked reduction of hepatic steatosis, without affecting body weight. These observations suggest a clinical implication of EPA for the treatment of NASH.

We previously reported that hCLS plays an important role in the progression from simple steatosis to NASH [8]. Since EPA treatment markedly suppressed hCLS formation as well as interstitial fibrosis in MC4R-KO mice, it is likely that one site of actions of EPA is hCLS. Given that CD11c-positive macrophages surround dead hepatocytes in hCLS, EPA may suppress hepatocyte injury and cell death. This notion is supported by our observations that EPA treatment effectively prevented the increase in the number of TUNEL-positive cells, serum ALT concentrations, and the hepatocyte injury score (ballooning) in MC4R-KO mice. Moreover, these effects were observed even when MC4R-KO mice were treated with EPA after NASH developed. During the development of NASH, hepatocytes store excessive lipid including toxic lipids (*i*.*e*. saturated free fatty acid, free cholesterol, and lysophosphatidyl choline), which leads to metabolic stress such as oxidative stress and endoplasmic reticulum stress, thereby activating the cell death program [1,36,37]. Notably, hepatocyte apoptosis is a prominent feature of human NASH as well [38]. In this regard, it is known that that *n-3* PUFAs can induce gene expression of ROS-degrading enzymes to inhibit oxidative stress, and antagonize the saturated fatty acid-induced endoplasmic reticulum stress [39,40]. It is, therefore, conceivable that EPA treatment ameliorates lipotoxicity of hepatocytes to prevent the formation of hCLS in MC4R-KO mice.

Fibrogenesis is a complex process that involves a variety of cells including both parenchymal cells and stromal cells like myofibroblasts and immune cells. Although EPA and *n-3* PUFAs are known to exert an anti-inflammatory property [10,41], EPA treatment did not affect the inflammation score and TNFα mRNA expression in the liver from MC4R-KO mice. TGFβ, a key regulator of fibrogenesis, is produced as a latent complex containing latency-associated protein and latent TGFβ binding protein, and then activated when released from the latent complex [30]. In this study, expression of uPAR, a cell surface receptor for plasma kallikrein, was increased in the liver of MC4R-KO mice, suggesting the plasma kallikrein-mediated TGFβ activation. Intriguingly, uPAR expression and active TGFβ levels were markedly suppressed by EPA treatment. Among several molecules that can activate TGFβ such as integrins, metalloproteinases and plasmin, plasma kallikrein plays an important role in animal models of liver fibrosis [34,42]. Since TNFα potently induces uPAR expression in cultured hepatic stellate cells [34], it is interesting to know how hCLS formation induces TGFβ activation during the development of NASH and how EPA suppresses the process.

We previously reported that complex interactions between adipose tissue and liver should play a role in the development of NASH in MC4R-KO mice [5]. For instance, unbalanced production of pro- and anti-inflammatory adipocytokines in obesity has been implicated in the pathogenesis of obesity-related complications including NASH [43]. Among numerous adipocytokines, there is substantial evidence on the protective role of adiponectin in the development of hepatic fibrosis and inflammation [44,45]. As a possible mechanism, adiponectin stimulates β-oxidation by activation of AMP-activated protein kinase and PPARα and down regulates expression of sterol regulatory element binding protein-1c to suppress *de novo* lipogenesis in the liver [46,47]. Adiponectin also exerts its inhibitory effect on platelet-derived growth factor BB- and TGFβ-induced proliferation and migration of hepatic stellate cells [48]. In this study, EPA treatment effectively increased the otherwise reduced serum adiponectin concentrations in MC4R-KO mice, which may be involved in the beneficial effect of EPA on liver injury. As we and others reported previously, serum adiponectin concentrations are elevated in obese mice and humans when treated with EPA or *n-3* PUFA-rich fish oil [26,49,50]. Moreover, we have provided evidence that EPA increases adiponectin secretion through the improvement of obesity-induced adipose tissue inflammation [26]. Neschen *et al*. also showed that fish oil activates PPARγ in adipocytes to increase adiponectin secretion [49]. On the other hand, EPA treatment did not show the effect on the serum concentrations of leptin in MC4R-KO mice, while it is known that leptin promotes liver fibrosis in certain liver fibrosis models [28,51,52]. Collectively, adipose tissue may contribute to the pathogenesis of liver fibrosis in MC4R-KO mice. It is, therefore, conceivable that the beneficial effect of EPA on liver injury of MC4R-KO mice is attributed to its action on adipose tissue as well as liver.

Clinical efficacy of *n-3* PUFAs for the treatment of NAFLD/NASH is still controversial. Tanaka *et al*. reported that highly purified EPA treatment improves biochemical and histological abnormalities in Japanese patients with NASH [21]. In contrast, a recent randomized, double-blind, placebo-controlled trial failed to prove the effectiveness of EPA for 12 months on hepatic steatosis and liver fibrosis in patients with NAFLD/NASH [22,23]. It is noteworthy that the dosage of EPA used in this study might not be enough for American population, where the well-established beneficial effect of EPA on dyslipidemia was not observed [22,23]. There was also no significant improvement in liver histology in several clinical trials using *n-3* PUFAs, in which the treatment showed only marginal effect on dyslipidemia [23–25]. Therefore, additional studies are required regarding the dosage of EPA and duration of the treatment. Moreover, dietary saturated fatty acid composition may affect the efficacy of EPA treatment, since experimental evidence in rodents suggest that EPA exerts its anti-inflammatory property, at least in part, through counteracting saturated fatty acids [26], and diet rich in saturated fatty acids augments insulin resistance and NAFLD [53,54]. Based on the heterogeneity of NAFLD/NASH patients, it is also important to evaluate the effect of EPA in subgroups with differential risk factors.

In conclusion, we demonstrate that EPA treatment effectively prevents the development and progression of liver fibrosis in MC4R-KO mice along with marked reduction of hepatic steatosis. EPA may exert its anti-fibrotic effect through suppression of hepatocyte injury-induced TGFβ activation in hCLS. Our data also suggest that EPA acts on adipose tissue as well as liver to ameliorate liver fibrosis. This study unravels a novel anti-fibrotic mechanism of EPA, thereby suggesting a clinical implication for the treatment of NASH.

## Supporting Information

S1 Fig Body weight and tissue weights in MC4R-KO mice in the therapeutic study. Body weight (A) and weights of the subcutaneous, epididymal, and mesenteric white adipose tissues (B) and liver (C) of male MC4R-KO before EPA treatment (Western diet (WD) supplemented with 5% (wt/wt) palmitate for 20 weeks) and after 4-week EPA treatment. MC4R-EPA Tx, MC4R-KO mice fed WD supplemented with 5% (wt/wt) EPA for 4 weeks after the development of NASH. (D) Liver triglyceride (TG) content at each time point. †† *P* < 0.01 vs. MC4R-control at 20 weeks; ** *P* < 0.01 vs. MC4R-control at 24 weeks; n.s., not significant. MC4R-control at 20 weeks, *n* = 9; MC4R-control at 24 weeks, *n* = 7; MC4R-EPA Tx, *n* = 10.
(TIF)

S2 Fig Hepatic mRNA expression in MC4R-KO mice in the therapeutic study. Hepatic mRNA expression levels after 4-week EPA treatment. mRNA expression of *de novo* lipogenesis (FAS and SCD-1) (fatty acid synthase (FAS) and stearoyl-CoA desaturase (SCD-1)) and β-oxidation (PPARα, CPT1A) (A), inflammatory markers (F4/80 and tumor necrosis factor α (TNFα) (B) and fibrogenic factors (transforming growth factor β1 (TGFβ1), collagen α1(I) (COL1A1), tissue inhibitor of metalloproteinase-1 (TIMP1), and matrix metalloproteinase-2 (MMP2)) (C). * *P* < 0.05; ** *P* < 0.01; n.s., not significant.
(TIF)

S3 Fig hCLS formation and TGFβ activation in the liver of MC4R-KO mice in the therapeutic study. (A) F4/80 immunostaining. Arrows indicate hepatic crown-like structures (hCLS). (B) Quantification of hCLS number after EPA treatment. (C) Immunofluorescent analysis for F4/80 and CD11c. (D) Hepatic mRNA expression of CD11c. Quantification of the TUNEL-positive cell number (E) and R58 LAP-DP-positive area (F). (G) Hepatic mRNA expression of urokinase-type plasminogen activator receptor (uPAR). (H) Active TGFβ1 protein levels in the liver. Scale bars, 50 μm. * *P* < 0.05; ** *P* < 0.01.
(TIF)

S1 Table Dietary composition of standard diet (CE-2) and Western diet (D12079B) used in this study. (DOCX)

S2 Table Primers used in this study. (DOCX)

S3 Table Fatty acid composition of the liver from MC4R-KO treated with EPA for 24 weeks. (DOCX)

S4 Table Serological parameters of MC4R-KO mice in the therapeutic study. (DOCX)
