# Supplementary figures and images for: Eicosapentaenoic Acid Ameliorates Non-Alcoholic Steatohepatitis in a Novel Mouse Model Using Melanocortin 4 Receptor-Deficient Mice
Source: PLoS One. 2015 Mar 27;10(3):e0121528. doi: 10.1371/journal.pone.0121528 (PMC4376873; doi:10.1371/journal.pone.0121528)

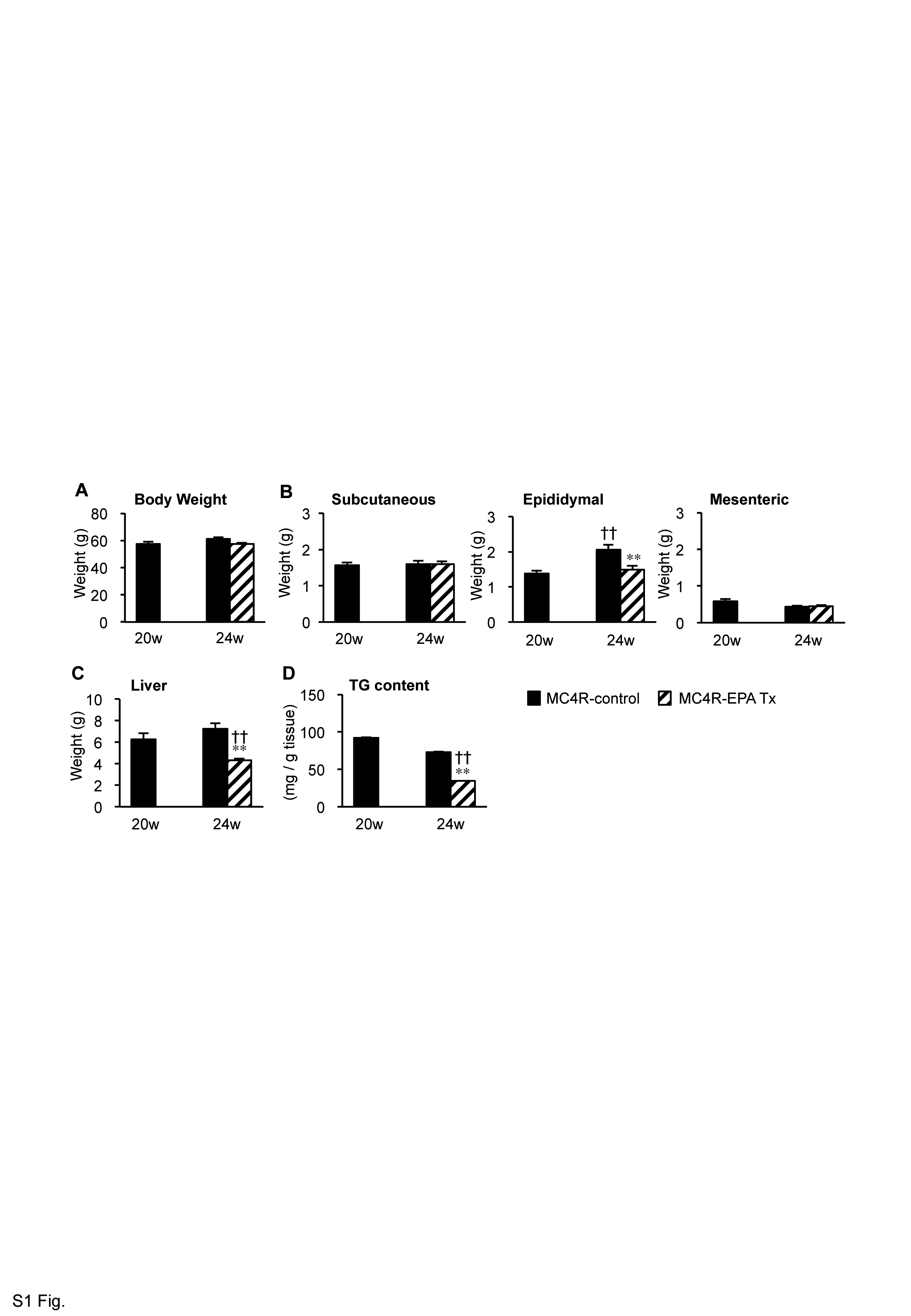

Supplement: S1 Fig — Body weight (A) and weights of the subcutaneous, epididymal, and mesenteric white adipose tissues (B) and liver (C) of male MC4R-KO before EPA treatment (Western diet (WD) supplemented with 5% (wt/wt) palmitate for 20 weeks) and after 4-week EPA treatment. MC4R-EPA Tx, MC4R-KO mice fed WD supplemented with 5% (wt/wt) EPA for 4 weeks after the development of NASH. (D) Liver triglyceride (TG) content at each time point. †† P < 0.01 vs. MC4R-control at 20 weeks; ** P < 0.01 vs. MC4R-control at 24 weeks; n.s., not significant. MC4R-control at 20 weeks, n = 9; MC4R-control at 24 weeks, n = 7; MC4R-EPA Tx, n = 10. (TIF) [file pone.0121528.s001.tif]

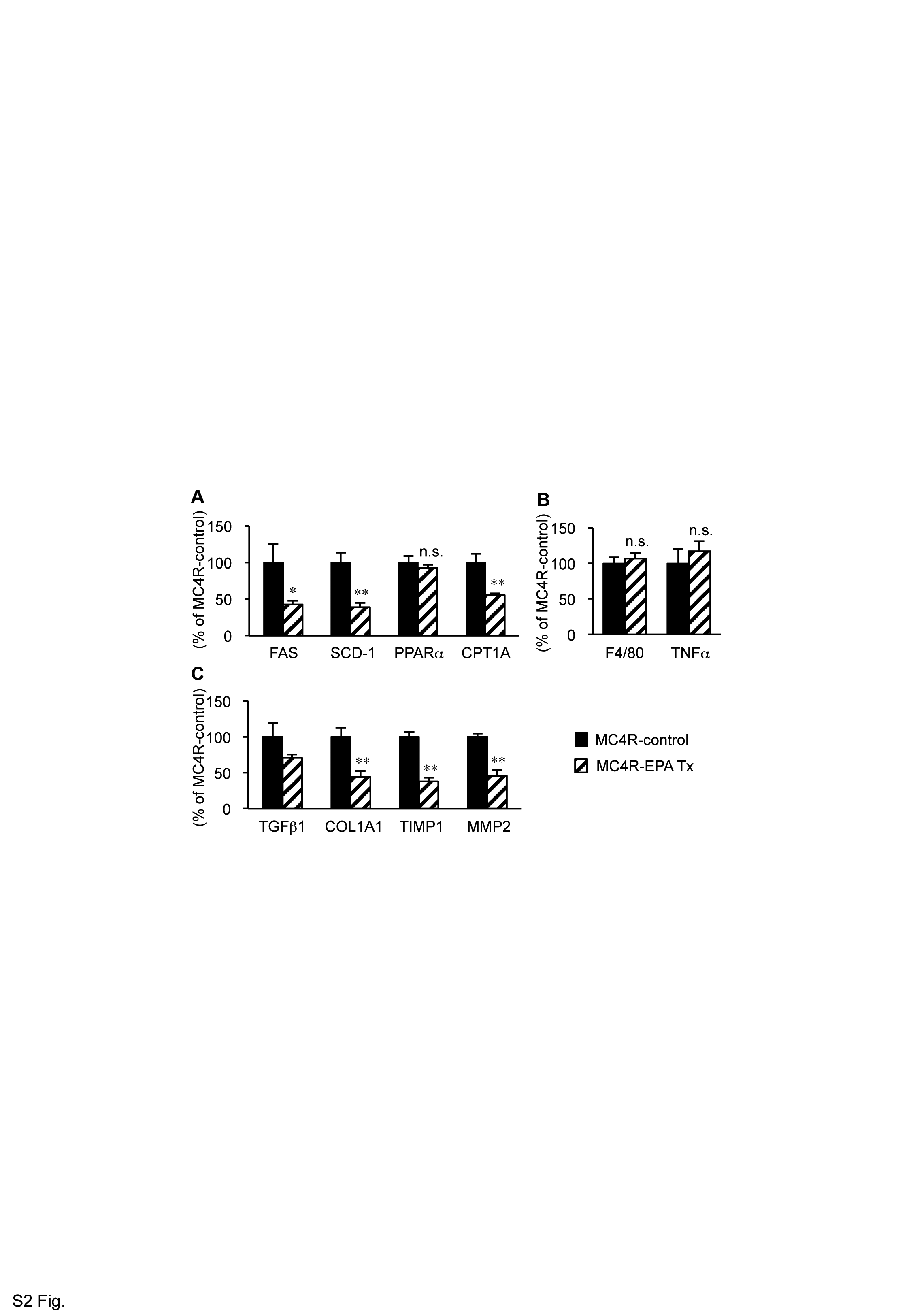

Supplement: S2 Fig — Hepatic mRNA expression levels after 4-week EPA treatment. mRNA expression of de novo lipogenesis (FAS and SCD-1) (fatty acid synthase (FAS) and stearoyl-CoA desaturase (SCD-1)) and β-oxidation (PPARα, CPT1A) (A), inflammatory markers (F4/80 and tumor necrosis factor α (TNFα) (B) and fibrogenic factors (transforming growth factor β1 (TGFβ1), collagen α1(I) (COL1A1), tissue inhibitor of metalloproteinase-1 (TIMP1), and matrix metalloproteinase-2 (MMP2)) (C). * P < 0.05; ** P < 0.01; n.s., not significant. (TIF) [file pone.0121528.s002.tif]

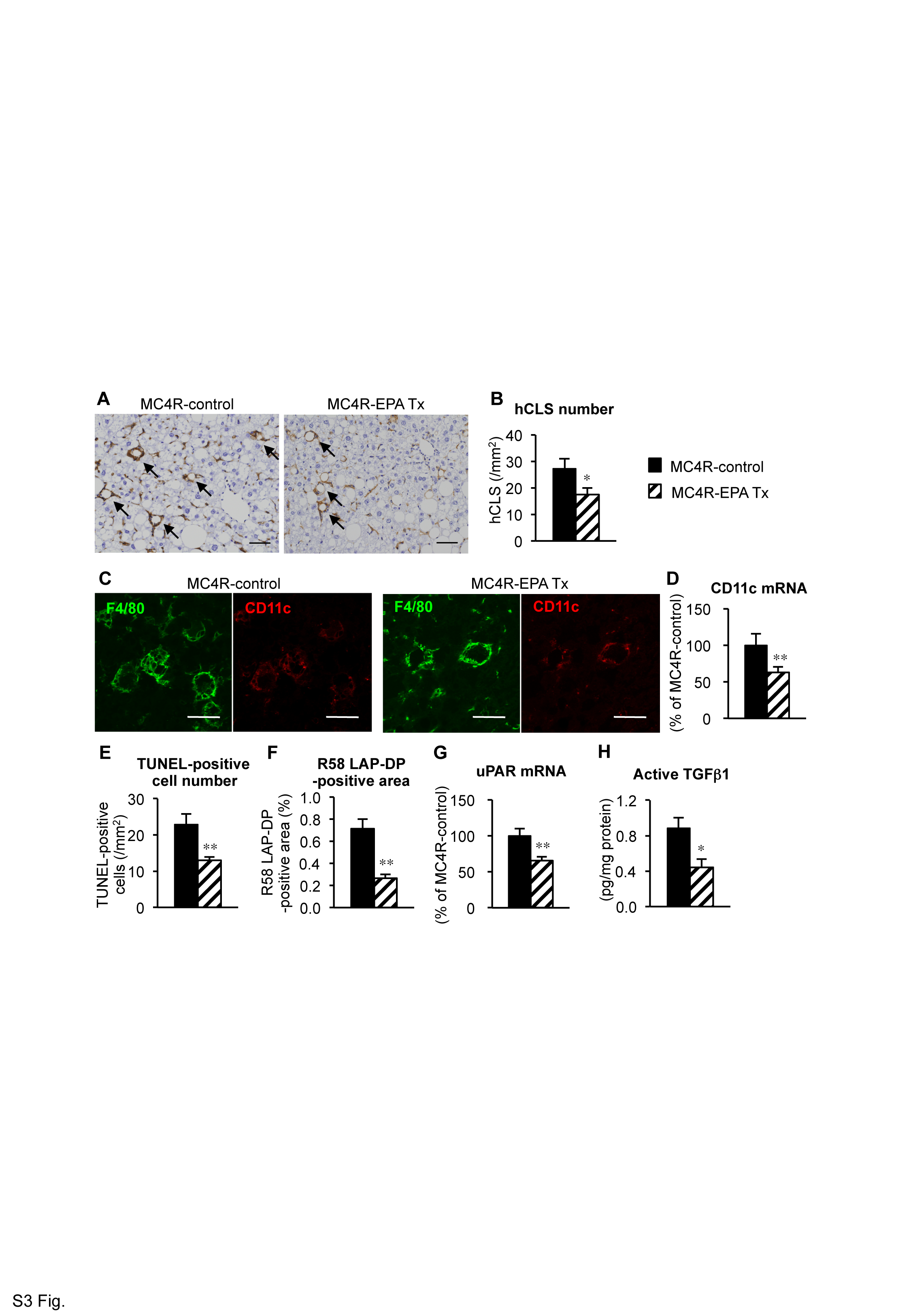

Supplement: S3 Fig — (A) F4/80 immunostaining. Arrows indicate hepatic crown-like structures (hCLS). (B) Quantification of hCLS number after EPA treatment. (C) Immunofluorescent analysis for F4/80 and CD11c. (D) Hepatic mRNA expression of CD11c. Quantification of the TUNEL-positive cell number (E) and R58 LAP-DP-positive area (F). (G) Hepatic mRNA expression of urokinase-type plasminogen activator receptor (uPAR). (H) Active TGFβ1 protein levels in the liver. Scale bars, 50 μm. * P < 0.05; ** P < 0.01. (TIF) [file pone.0121528.s003.tif]
