# Supplementary material for: Eicosapentaenoic Acid Ameliorates Non-Alcoholic Steatohepatitis in a Novel Mouse Model Using Melanocortin 4 Receptor-Deficient Mice
Source: PLoS One. 2015 Mar 27;10(3):e0121528. doi: 10.1371/journal.pone.0121528 (PMC4376873; doi:10.1371/journal.pone.0121528)
Supplement: S1 Table — (DOCX) [file pone.0121528.s004.docx]

**S1 Table. Dietary composition of standard diet (CE-2) and Western diet (D12079B) used in this study.**

CE-2 D12079B

Protein (kcal %) 29.2 17.0

Carbohydrate (kcal %) 58.2 43.0

Fat (kcal %) 12.6 41.0

Fatty acid composition (% (wt/wt) of total fatty acids)

C2:0, Acetic N.D. N.D.

C4:0, Butyric N.D 3.3

C6:0, Caproic N.D. 1.9

C8:0, Caprylic N.D. 1.1

C10:0, Capric N.D. 2.5

C12:0, Lauric N.D. 2.8

C14:0, Myristic 0.8 10.2

C14:1, Myristoleic N.D. 1.5

C16:0, Palmitic 16.3 27.2

C16:1, Palmitoleic 1.3 2.3

C17:0, Heptadecanoic 0.5 N.D.

C18:0, Stearic 2.1 12.4

C18:1, Oleic 21.0 26.8

C18:1, Vaccenic 2.1 N.D.

C18:1, Elaidic 2.1 N.D.

C18:2, Linoleic 44.4 5.4

C18:3, Linolenic 3.3 1.5

C20:0, Arachidic 0.5 1.0

C20:1, Eicosenoic 0.7 N.D.

C20:4, Arachidonic N.D. N.D.

C20:5, Eicosapentaenoic 2.3 N.D.

C22:0, Behenic 0.3 N.D.

C22:5, Dicisaoebtaenoic N.D. N.D.

C22:6, Docosahexaenoic 1.3 N.D.

C24:0, Lignoceric 0.2 N.D.

C24:1, Tetracosenoic 0.2 N.D.

Saturated (%) 20.6 62.5

Monounsaturated (%) 25.3 30.7

Polyunsaturated (%) 51.3 6.9

N.D., not detected
