# Supplementary material for: Eicosapentaenoic Acid Ameliorates Non-Alcoholic Steatohepatitis in a Novel Mouse Model Using Melanocortin 4 Receptor-Deficient Mice
Source: PLoS One. 2015 Mar 27;10(3):e0121528. doi: 10.1371/journal.pone.0121528 (PMC4376873; doi:10.1371/journal.pone.0121528)
Supplement: S2 Table — (DOCX) [file pone.0121528.s005.docx]

**S2 Table. Primers used in this study.**

Genes Primers

CD11c Fw: 5’-GCCATTGAGGGCACAGAGA-3’

Rv: 5’-GAAGCCCTCCTGGGACATCT-3’

COL1A1 Fw: 5’-CCTCAGGGTATTGCTGGACAAC-3’

Rv: 5’-ACCACTTGATCCAGAAGGACCTT-3’

CPT1A Fw: 5’-CCTGCATTCCTTCCCATTTG-3’

Rv: 5’-TGCCCATGTCCTTGTAATGTG-3’

F4/80 Fw: 5’-CTTTGGCTATGGGCTTCCAGT-3’

Rv: 5’-GCAAGGAGGACAGAGTTTATCGTG-3’

FAS Fw: 5’-CCTGGATAGCATTCCGAACCT-3’

Rv: 5’-AGCACATCTCGAAGGCTACACA-3’

MMP2 Fw: 5’-CCCCATGAAGCCTTGTTTACC-3’

Rv: 5’-TTGTAGGAGGTGCCCTGGAA-3’

PPARα Fw: 5’-AGGAAGCCGTTCTGTGACAT-3’

Rv: 5’-AATCCCCTCCTGCAACTTCT-3’

SCD-1 Fw: 5’-CCGGAGACCCCTTAGATCGA-3’

Rv: 5’-TAGCCTGTAAAAGATTTCTGCAAACC-3’

TGFβ1 Fw: 5’-CCTGAGTGGCTGTCTTTTGACG-3’

Rv: 5’-AGTGAGCGCTGAATCGAAAGC-3’

TIMP1 Fw: 5’-CATCACGGGCCGCCTA-3’

Rv: 5’-AAGCTGCAGGCACTGATGTG-3’

TNFα Fw: 5’-ACCCTCACACTCAGATCATCTTC-3’

Rv: 5’-TGGTGGTTTGCTACGACGT-3’

uPAR Fw: 5’-GCCGCTATCCTACAGAGCAC-3’

Rv: 5’-GCTATGGAAACCTGCTGTGCC-3’

36B4 Fw: 5’-GGCCCTGCACTCTCGCTTTC-3’

Rv: 5’-TGCCAGGACGCGCTTGT-3’
