# Supplementary material for: Eicosapentaenoic Acid Ameliorates Non-Alcoholic Steatohepatitis in a Novel Mouse Model Using Melanocortin 4 Receptor-Deficient Mice
Source: PLoS One. 2015 Mar 27;10(3):e0121528. doi: 10.1371/journal.pone.0121528 (PMC4376873; doi:10.1371/journal.pone.0121528)
Supplement: S3 Table — (DOCX) [file pone.0121528.s006.docx]

**S3 Table. Fatty acid composition of the liver from MC4R-KO treated with EPA for 24 weeks.**

WT MC4R-KO

(% of total fatty acid) SD Control EPA Pre

C12:0, Lauric 0.05 ± 0.01 0.07 ± 0.00 0.05 ± 0.00

C14:0, Myristic 0.28 ± 0.01 0.69 ± 0.04^**^ 0.62 ± 0.04

C16:0, Palmitic 26.94 ± 0.46 24.27 ± 0.69 22.79 ± 0.92

C16:1, Palmitoleic 2.77 ± 0.10 8.70 ± 0.60^**^ 5.97 ± 0.13 ^¶^

C18:0, Stearic 9.01 ± 0.29 5.27 ± 0.15^**^ 8.37 ± 0.33^¶^

C18:1, Oleic 15.94 ± 0.45 36.13 ± 0.54^**^ 28.69 ± 1.48^¶^

C18:1, Vaccenic 3.07 ± 0.11 9.42 ± 0.35^**^ 2.58 ± 0.32^¶^

C18:2, Linoleic 17.54 ± 0.50 4.25 ± 0.50^**^ 5.29 ± 0.33

C18:3, γ-Linolenic 0.26 ± 0.01 0.08 ± 0.01^**^ 0.08 ± 0.01

C18:3, α-Linolenic 0.35 ± 0.03 0.02 ± 0.00^**^ 0.20 ± 0.02^¶^

C20:0, Arachidic 0.21 ± 0.01 0.05 ± 0.01^**^ 0.12 ± 0.02^¶^

C20:1, Eicosenoic 0.39 ± 0.02 0.66 ± 0.05^**^ 0.28 ± 0.02^¶^

C20:2, Eicosadienoic 0.26 ± 0.01 0.06 ± 0.00^**^ 0.04 ± 0.00

C20:3, Dihomo-γ-linolenic 1.65 ± 0.07 1.14 ± 0.12^**^ 0.33 ± 0.04^¶^

C20:4, Arachidonic 7.95 ± 0.28 6.07 ± 0.14^**^ 1.09 ± 0.05^¶^

C20:5, Eicosapentaenoic 1.51 ± 0.07 0.14 ± 0.01 14.95 ± 1.49^¶^

C22:0, Behenic 0.09 ± 0.02 0.04 ± 0.00 0.08 ± 0.01

C22:4, Adrenic 0.13 ± 0.01 0.17 ± 0.01^*^ 0.01 ± 0.00^¶^

C22:5, Docosapentaenoic 1.02 ± 0.03 0.11 ± 0.01 4.70 ± 0.40^¶^

C24:0, Lignoceric 0.08 ± 0.01 0.03 ± 0.00^**^ 0.02 ± 0.00

C22:6, Docosahexaenoic 10.38 ± 0.35 2.46 ± 0.01^**^ 3.58 ± 0.69

Data are expressed as the mean ± SE. ^**^*P* < 0.01 vs. WT-SD; ^¶^ *P* < 0.01 vs. MC4R-Control. *n* = 4.
