# Supplementary material for: Eicosapentaenoic Acid Ameliorates Non-Alcoholic Steatohepatitis in a Novel Mouse Model Using Melanocortin 4 Receptor-Deficient Mice
Source: PLoS One. 2015 Mar 27;10(3):e0121528. doi: 10.1371/journal.pone.0121528 (PMC4376873; doi:10.1371/journal.pone.0121528)
Supplement: S4 Table — (DOCX) [file pone.0121528.s007.docx]

**S4 Table. Serological parameters of MC4R-KO mice in the therapeutic study.**

MC4R-KO

Control EPA Tx

BG (* lib*, mg/dL) 127.4 ± 6.7 142.8 ± 13.0

TG (mg/dL) 35.7 ± 2.3 28.6 ± 2.9

TC (mg/dL) 288.4 ± 24.0 169.4 ± 12.7^**^

FFA (mEq/L) 1.13 ± 0.09 0.96 ± 0.06

ALT (IU/L) 658.0 ± 225.6 336.1 ± 95.0^*^

Adiponectin (μg/mL) 8.1 ± 0.7 14.8 ± 1.2^**^

Leptin (ng/mL) 101.0 ± 15.7 81.8 ± 5.0

BG, blood glucose; TG, triglyceride; FFA, free fatty acid; TC, total cholesterol; ALT, alanine aminotransferase.

Data are expressed as the mean ± SE. ^*^ *P* < 0.05, ^**^*P* < 0.01. *n* = 7-10.
